# Hepatitis A Virus Infection and Molecular Research

**DOI:** 10.3390/ijms23137214

**Published:** 2022-06-29

**Authors:** Tatsuo Kanda, Reina Sasaki-Tanaka, Shingo Nakamoto

**Affiliations:** 1 Division of Gastroenterology and Hepatology, Department of Medicine, Nihon University School of Medicine, 30-1 Oyaguchi-kamicho, Itabashi-ku, Tokyo 173-8610, Japan; sasaki.reina@nihon-u.ac.jp; 2 Department of Gastroenterology, Graduate School of Medicine, Chiba University, 1-8-1 Inohana, Chuo-ku, Chiba 260-8670, Japan; nakamotoer@faculty.chiba-u.jp

Hepatitis A virus (HAV) infection is a major cause of acute viral hepatitis globally, which can occasionally lead to acute liver failure (ALF) and acute-on-chronic liver failure (ACLF), which often result in death without liver transplantation. Along with concomitant increases in alcohol misuse and metabolic syndrome in recent years, HAV plays a role as an acute condition in patients with ACLF [1]. Although HAV vaccination could prevent people from being infected with HAV, HAV vaccination frequency is too low to prevent HAV infection, at least in Japan. As the number of people without anti-HAV immunity is increasing in the Asian Pacific region, according to the improvement of public health, it is important to develop anti-HAV drugs and to distribute HAV vaccinations [2]. HAV RNA genome consists of a single, long, open reading frame (ORF) flanked by a 5′-untranslated region (UTR) and 3′-UTR. The HAV internal ribosomal entry site (IRES) is located in 5′-UTR, and translates as HAV proteins in a cap-independent manner. ORF encodes structural (VP4, VP2, VP3, VP1, and pX) and non-structural (2B, 2C, 3A, 3B, 3C, and 3D) proteins [3]. One large immature protein, encoded by ORF, is primarily cut into at least ten mature proteins by HAV 3C protease, and HAV 3D has an RNA-dependent RNA polymerase, which is important for HAV replication. HAV IRES, 3C protease, and 3D polymerase in HAV genome or HAV protein are attractive targets of anti-HAV drugs (Figure 1) [4,5,6,7,8,9,10,11].

Zinc compounds suppress HAV replication [13,14]. In this Special Issue, Kanda et al. [15] revealed that zinc chloride inhibits mitogen-activated protein kinase 3 (MAP2K3) expression and downregulates HAV replication in human hepatocytes. The inhibition of MAP2K3 may also prevent patients infected with HAV from developing ALF and ACLF.

Sasaki-Tanaka et al. [9] examined several novel therapeutic drugs through a drug repositioning approach, targeting RNA-dependent RNA polymerase and RNA-dependent DNA polymerase. They found that favipiravir effectively suppressed HAV replication through the introduction of mutagenesis into the HAV genome. Of interest is that favipiravir was effective in reducing the replication of severe acute respiratory syndrome coronavirus 2 (SARS-CoV-2) [16].

Sasaki-Tanaka et al. [10] performed the in silico screening of anti-HAV compounds targeting the HAV 3C protease enzyme, using the Schrodinger Glide molecular docking program to study the interactions between the ligand and HAV 3C protease enzyme at an atomic level, from an antiviral library of 25,000 compounds, to evaluate anti-HAV 3C protease inhibitors. They found the HAV 3C protease inhibitor Z10325150 (Enamine, Kyiv, Ukraine) and confirmed the HAV replication inhibitory activity in human hepatocytes.

In this Special Issue, Tréguier et al. [17] also reviewed the crucial role of apolipoprotein E (ApoE) in the lifecycles of hepatitis B virus (HBV) and hepatitis C virus (HCV) and discussed its potential role in the lifecycle of other hepatotropic viruses, including HAV. Hepatotropic viruses infect hepatocytes and spread throughout the liver, using ApoE, which seems one of the attractive targets of anti-HAV drugs.

HCV NS5A activates the glucokinase (GCK) isoenzyme of hexokinases through its D2 domain (NS5A-D2) [18]. HCV NS5A-D2 can reprogram central carbon metabolism towards a more energetic and glycolytic phenotype compatible with the needs of HCV for replication. Knockout of these host factor genes may impair HCV replication [19].

Lesnova et al. [20] demonstrated the first evidence of immunomodulatory activity of antioxidant N-acetylcysteine (NAC) and polyamine biosynthesis inhibitor 2-difluoromethylornithine (DFMO) during prophylactic immunization against infectious diseases. NAC and DFMO may be used as new adjuvant compounds that enhance the immune response, and it might be useful for the development of vaccines against hepatitis viruses.

In summary, this Special Issue offers a critical overview of recent research in hepatitis A virus and molecular research, and the related areas.

## Figures and Tables

**Figure 1:**
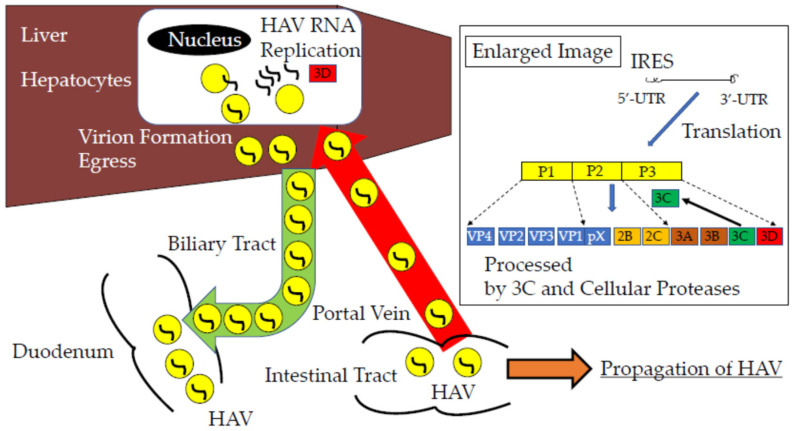
Hepatitis A virus (HAV) infection into hepatocytes of the liver. HAV internal ribosomal entry-site (IRES), 3C protease, and 3D ribonucleic acid (RNA)-dependent RNA polymerase are attractive targets of anti-HAV drugs [11]. HAV 5′-untranslated region (UTR) has conservative RNA sequences among different strains [8,12].

## Data Availability

Data might be available from the authors of the cited papers.
